# Spontaneous passage of an accidentally ingested metallic bullet casing in an adolescent: a case report

**DOI:** 10.1186/s12245-025-01047-3

**Published:** 2025-11-05

**Authors:** Sowdo Nur Iyow, Abdullahi Ahmed Ahmed, Abdulkadir Nur Mohamed, Shuayb Moallim Ali Jama, Hassan Adan Ali Adan

**Affiliations:** 1 Department of Emergency Medicine, Mogadishu Somali Turkiye Recep Tayyip Erdogan Training and Research Hospital, Digfer Road, Hodon, Mogadishu, Somalia; 2 Department of General Surgery, Mogadishu Somali Turkiye Recep Tayyip Erdogan Training and Research Hospital, Mogadıshu, Somalia; 3 Department of Radiology, Mogadishu Somali Turkiye Recep Tayyip Erdogan Training and Research Hospital, Mogadıshu, Somalia

**Keywords:** Bullet casing, Foreign body ingestion, Adolescent, Conservative management, Gastrointestinal tract, Emergency department

## Abstract

**Background:**

Accidental ingestion of foreign objects is a frequent emergency presentation that can lead to serious complications. It is most often seen in children and high-risk adults, but remains rare in healthy adolescents without predisposing factors.

**Case presentation:**

We describe a 17-year-old female who accidentally swallowed a metallic bullet casing during a meal. She presented two hours later with only a mild sore throat. Serial abdominal radiographs demonstrated progressive passage of the casing through the gastrointestinal tract without perforation or obstruction. With close inpatient monitoring, including clinical evaluations and sequential imaging, the casing was spontaneously expelled per rectum. The patient remained asymptomatic, and follow-up showed no complications.

**Discussion:**

Most blunt, small foreign bodies pass spontaneously. This case highlights that, in carefully selected asymptomatic patients, conservative management with vigilant monitoring is a safe and effective alternative to invasive intervention.

**Conclusion:**

Conservative management can be both safe and effective in healthy adolescents who accidentally ingest a foreign body, provided there are no clinical or radiological signs of obstruction, perforation, or other complications.

## Introduction

Foreign body ingestion is a common clinical issue encountered in emergency practice, accounting for significant morbidity and an estimated 1,500 deaths annually in the United States [1]. It is most frequently seen in children, as well as in adults with underlying risk factors such as alcohol use, edentulism, psychiatric illness, or gastrointestinal pathology [1, 2]. In adults, conditions such as strictures (about 37%), malignancies (10%), esophageal rings (6%), and achalasia (2%) are commonly associated [3]. Although it is uncommon in teenagers, ingestion by accident can nonetheless happen to otherwise healthy people who do not have any risk factors.

Imaging is essential for both diagnosis and treatment. While CT can offer more information, especially in cases of suspected perforation, abdominal radiographs are usually employed to locate and describe swallowed foreign items [4]. While endoscopic or surgical intervention is reserved for high-risk instances, such as sharp objects, batteries, magnets, or those surpassing established size standards, the majority of ingested objects that are small, dull, and smooth pass on their own [5, 6].

Here, we describe a rare instance of a teenager inadvertently swallowing a metallic bullet casing, which was effectively treated conservatively. This case emphasizes how crucial cautious patient selection and close observation are to ensuring safe outcomes.

## Case presentation

A 17-year-old female presented to the emergency department two hours after inadvertently swallowing an elongated metallic bullet casing while eating dinner. She reported being distracted by her mobile phone when she suddenly felt herself swallow a hard metallic object. She denied abdominal pain, nausea, vomiting, rectal bleeding, choking, coughing, chest pain, or shortness of breath, though she did complain of mild throat discomfort. Her medical, surgical, psychiatric, and family history were unremarkable, and she denied substance or drug use.

On examination, her vital signs were stable. There were no dental abnormalities, and chest, abdominal, and digital rectal examinations were normal. Hematological investigations were within normal limits.

**Fig. 1:**
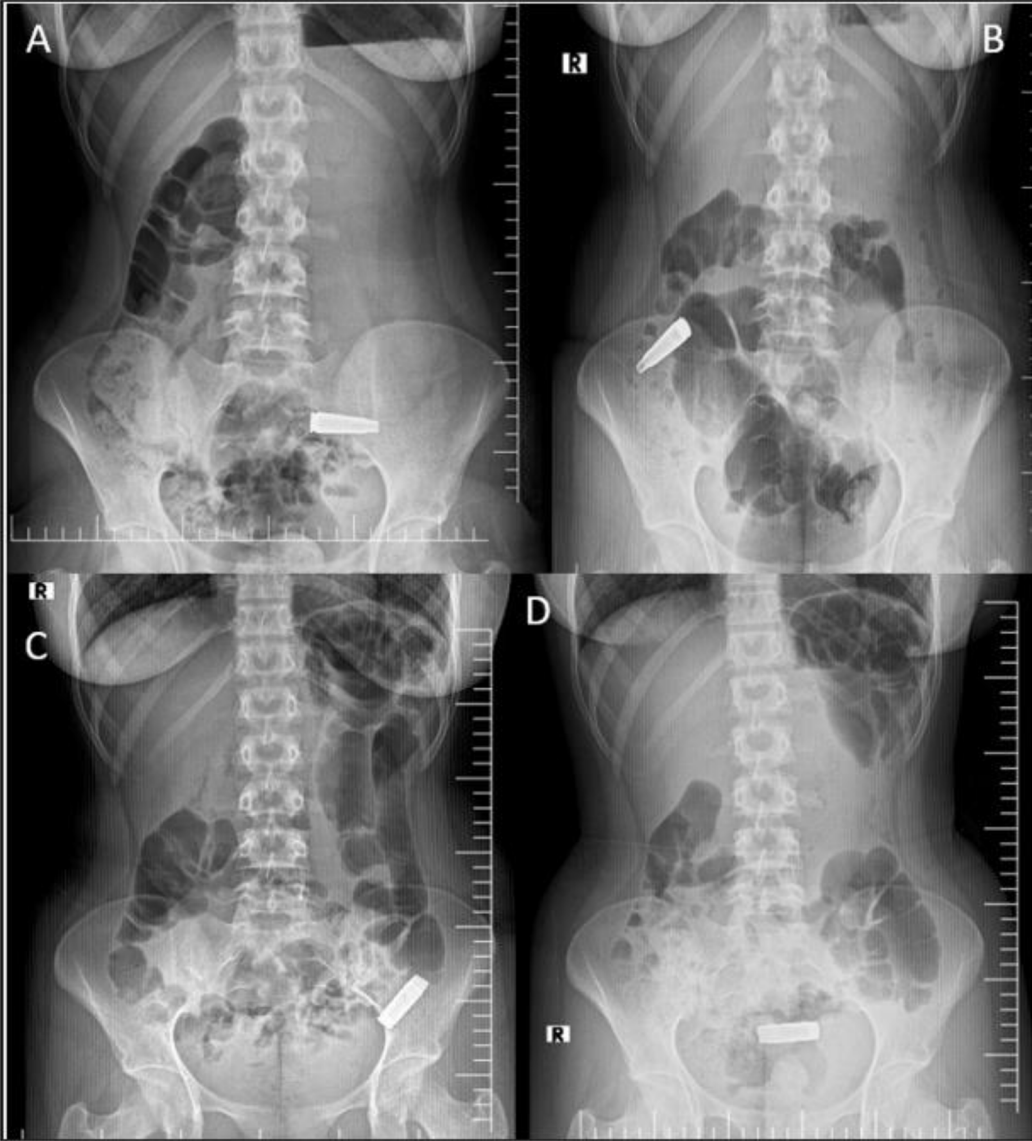
Sequential abdominal radiographs, demonstrated the progressive transit of a bullet shell casing through the gastrointestinal tract, migrating from the terminal ileum (**A**), to proximal ascending colon (**B**), to distal descending colon (**C**) and to the rectum (**D**)

At 3 h post-ingestion, an upright abdominal radiograph showed a radiopaque, elongated bullet casing positioned obliquely in the left lower quadrant, consistent with the terminal ileum (Fig. 1A). There was no radiographic or clinical indication of a perforation, and the patient remained stable. After being admitted to the surgical ward, she was closely monitored with repeat imaging, vital sign surveillance, and serial abdominal examinations.

A follow-up radiograph taken 11 h after ingestion showed that the bullet casing had migrated to the right lower abdomen, which corresponds to the proximal ascending colon (Fig. 1B). A follow-up radiograph taken 22 h after ingestion showed additional progression to the distal descending colon (Fig. 1C). The shell was visible in the rectum 28 h after consumption (Fig. 1D).

**Fig. 2:**
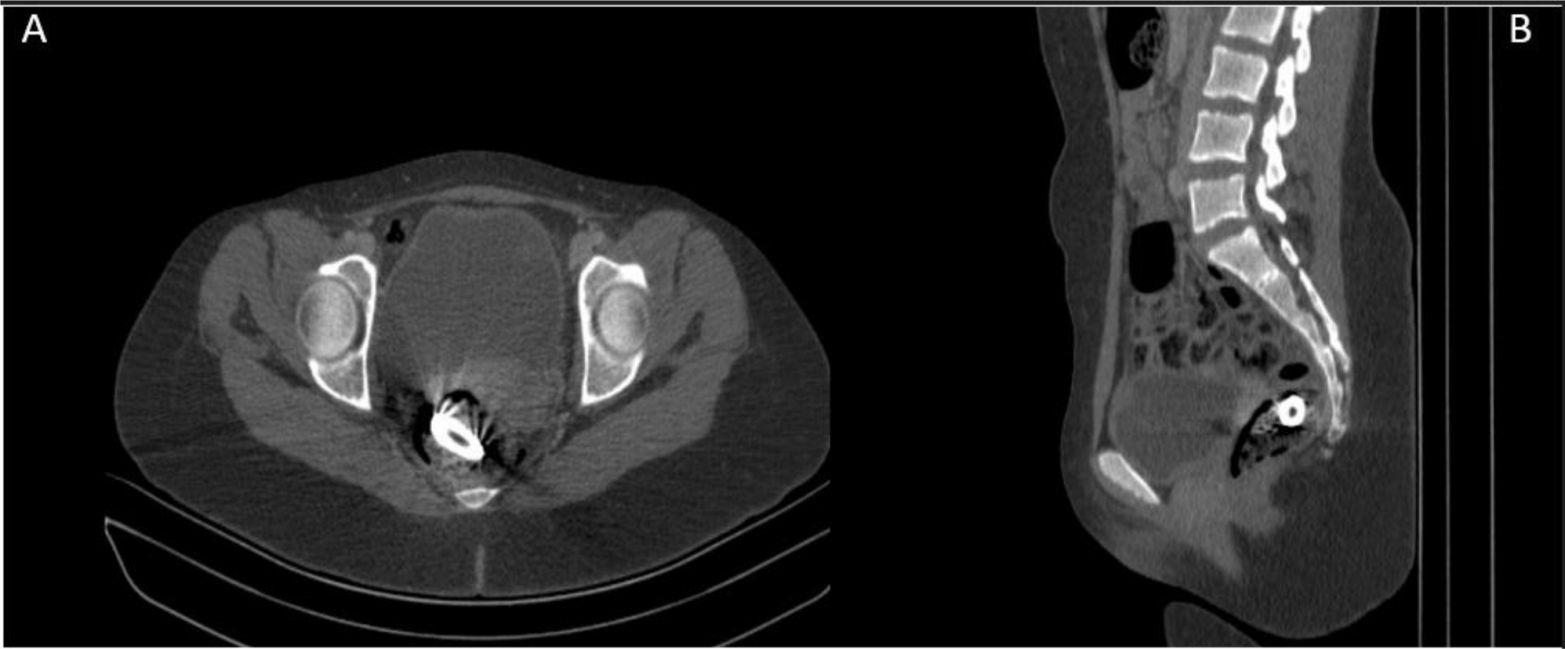
Axial (**A**) and sagittal (**B**) abdominopelvic CT images demonstrate a bullet shell casing lodged in the distal rectum, without evidence of bowel perforation

Although the foreign body had reached the rectum, a contrast-enhanced abdominopelvic computed tomography (CT scan) was performed at 39 h post-ingestion to better delineate its exact position and to exclude potential complications such as mucosal injury or perforation. Contrast-enhanced CT is indicated when complications such as obstruction, perforation, or migration of the foreign body are suspected, or when the object is radiolucent and not clearly visible on plain radiographs. Axial and sagittal pelvic CT images confirmed the casing lodged within the distal rectum, without evidence of free air, perforation, or obstruction. (Fig. 2A&B).

**Fig. 3:**
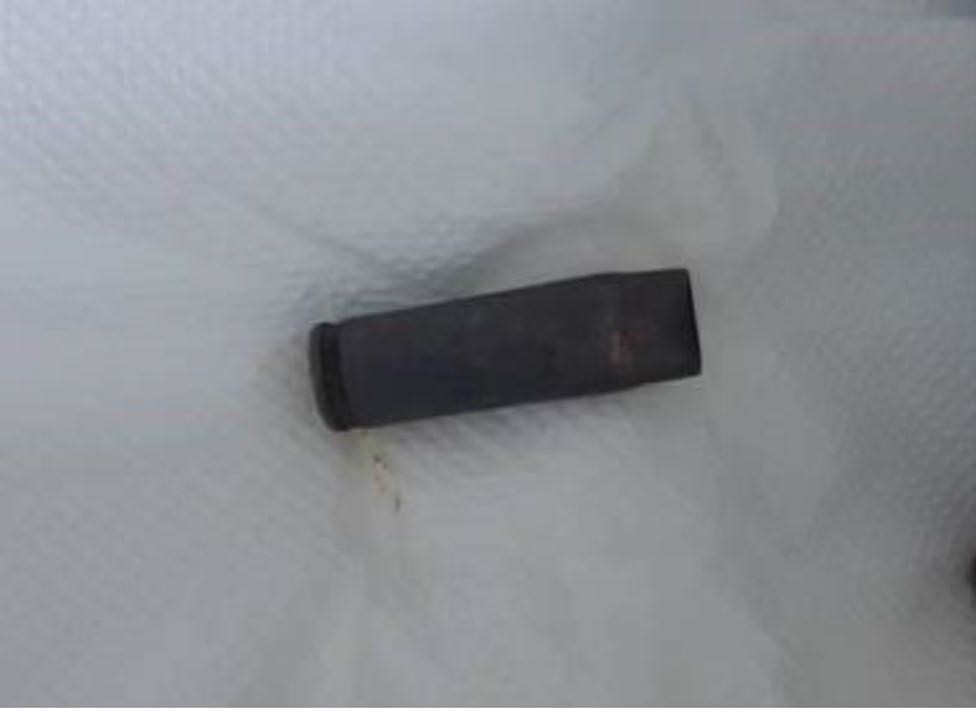
The expelled metallic bullet casing, approximately 2.5 cm × 0.7 cm, Y-shaped in appearance, was spontaneously expelled through the rectum without any intervention

At 49 h post-ingestion, the patient spontaneously expelled the bullet casing per rectum without intervention (Fig. 3). During a further 24 h of inpatient surveillance, she had no symptoms and her vital signs and abdominal results stayed stable.

She was discharged in good condition and reviewed six weeks later in the surgical clinic, where her examination was unremarkable. At a six-month telephone follow-up, she reported complete recovery and had resumed normal daily activities with no complications.

## Discussion

Foreign body ingestion is a relatively common clinical problem that can occasionally be life-threatening [1]. In the United States, approximately 120,000 cases are reported annually, most of which occur in children between 6 months and 3 years of age [7]. In adults, high-risk groups include individuals with acute intoxication, severe psychiatric illness, intellectual disabilities, or those seeking secondary gain, such as incarcerated persons [8]. Accidental bolus ingestion is also frequently observed in the elderly, particularly those who are edentulous and unable to chew solid foods adequately. Such patients often have underlying pathologies, including strictures, malignancies, achalasia, or esophageal rings, which predispose them to swallowing difficulties [2, 9].

Clinical presentation varies depending on the type and location of the foreign body and the duration of retention. Some patients remain asymptomatic, while others present with abdominal discomfort, nausea, vomiting, hematemesis, rectal bleeding, fever, or diarrhea [10]. Our patient was an otherwise healthy adolescent with no identifiable risk factors, whose only symptom was transient throat discomfort after accidentally swallowing a metallic bullet casing.

Radiography remains the primary diagnostic tool for detecting radiopaque foreign bodies, helping to determine their size, number, location, orientation, and shape, as well as the presence of sharp edges [4, 11]. It also allows monitoring of progression and can detect free air suggestive of perforation. However, small or radiolucent materials, including thin metals, wood, or glass, may be difficult for conventional radiography to detect [12]. Fish bones at restricted or angulated positions cause most gastrointestinal perforations. As many as 83% affect the ileum [13].

Clinical stability, complications, and foreign body kind and location affect care. Sharp items, batteries, and high-risk instances should be removed endoscopically or surgically to avoid perforation, bleeding, or infection [6, 14, 15]. Blunt objects shorter than 6 cm in length or narrower than 2.5 cm in diameter usually pass naturally; therefore, conservative observation is advised [16]. Foreign bodies lodged in the upper gastrointestinal tract or not moving past the stomach for more than a week, especially at fixed anatomical places like the ileocecal junction, may require intervention [1, 17].

Our case indicates that conservative treatment with imaging and periodic clinical examinations can be safe and effective for asymptomatic adults without neurodevelopmental or psychiatric issues. Importantly, these people can accurately report new symptoms, allowing timely treatment if issues arise. Choosing patients carefully, taking extensive medical histories, monitoring constantly, and having a low escalation threshold are crucial when utilizing a conservative strategy.

## In conclusion

When treating patients who inadvertently swallow foreign objects like gunshot casings, clinicians should weigh the advantages of cautious, conservative treatment against the necessity of prompt action. While advanced imaging or invasive methods should be saved for certain situations, plain radiography is still a useful, non-invasive method for locating and tracking radiopaque items. Regular clinical evaluation, grounded in thorough history-taking and physical examination is essential to detect complications early and to guide safe, individualized management.

## Data Availability

The data that support the findings of this study are available in Mogadishu Somali Turkey, Recep Tayyip Erdogan Training and Research Hospital information system. Data are however allowed to the authors upon reasonable request and with permission of the education and research committee.
